# Effects of aflibercept and bevacizumab on cell viability, cell metabolism and inflammation in hypoxic human Müller cells

**DOI:** 10.1371/journal.pone.0300370

**Published:** 2024-03-27

**Authors:** Monique Matsuda, Rafael André da Silva, Vinicius Moraes de Paiva Roda, Mônica Valéria Marquezini, Mário Luiz Ribeiro Monteiro, Dânia Emi Hamassaki

**Affiliations:** 1 Laboratory of Investigation in Ophthalmology (LIM-33), Division of Ophthalmology, School of Medicine, University of São Paulo, São Paulo, SP, Brazil; 2 Department of Cell & Developmental Biology, Institute of Biomedical Sciences, University of São Paulo, São Paulo, SP, Brazil; University of Rochester FEI: University of Rochester David and Ilene Flaum Eye Institute, UNITED STATES

## Abstract

Anti-VEGF (vascular endothelial growth factor) drugs such as aflibercept (AFL) and bevacizumab (BVZ) inhibit pathological neo-angiogenesis and vascular permeability in retinal vascular diseases. As cytokines and growth factors are produced by Müller glial cells under stressful and pathological conditions, we evaluated the *in vitro* effect of AFL (Eylea^®^, 0.5 mg/mL) and BVZ (Avastin^®^, 0.5 mg/mL) on cell viability/metabolism, and cytokine/growth factor production by Müller cells (MIO-M1) under cobalt chloride (CoCl_2_)-induced hypoxia after 24h, 48h and 72h. Cell viability/metabolism were analyzed by Trypan Blue and MTT assays and cytokine/growth factors in supernatants by Luminex xMAP-based multiplex bead-based immunoassay. Cell viability increased with AFL at 48h and 72h and decreased with BVZ or hypoxia at 24h. BVZ-treated cells showed lower cell viability than AFL at all exposure times. Cell metabolism increased with AFL but decreased with BVZ (72h) and hypoxia (48h and72h). As expected, AFL and BVZ decreased VEGF levels. AFL increased PDGF-BB, IL-6 and TNF-α (24h) and BVZ increased PDGF-BB (72h). Hypoxia reduced IL-1β, -6, -8, TNF-α and PDGF-BB at 24h, and its suppressive effect was more prominent than AFL (EGF, PDGF-BB, IL-1β, IL-6, IL-8, and TNF-α) and BVZ (PDGF-BB and IL-6) effects. Hypoxia increased bFGF levels at 48h and 72h, even when combined with anti-VEGFs. However, the stimulatory effect of BVZ predominated over hypoxia for IL-8 and TNF-α (24h), as well as for IL-1β (72h). Thus, AFL and BVZ exhibit distinct exposure times effects on MIO-M1 cells viability, metabolism, and cytokines/growth factors. Hypoxia and BVZ decreased MIO-M1 cell viability/metabolism, whereas AFL likely induced gliosis. Hypoxia resulted in immunosuppression, and BVZ stimulated inflammation in hypoxic MIO-M1 cells. These findings highlight the complexity of the cellular response as well as the interplay between anti-VEGF treatments and the hypoxic microenvironment.

## Introduction

Vascular endothelial growth factor (VEGF) is a crucial molecule in the development and upkeep maintenance of normal vascularization, as well as pathological neo-angiogenesis and vascular permeability in retinal vascular diseases. Vascular anomalies during the course of the disease results in opacity, fluid leakage, inflammation, and structural tissue damage [1]. Exacerbated VEGF signaling has been associated with the progression of diabetic retinopathy, retinal vascular occlusions, retinopathy of prematurity and age-related macular degeneration [2].

Anti-VEGF agents are commonly utilized in ophthalmic clinics to treat neovascularization in retinal vascular diseases, and their efficacy has been documented in several independent phase-III clinical trials [3–5]. However, despite their ability to diminish retinal vascularization and vascular leakage, VEGF inhibition agents such as aflibercept, ranibizumab, pegaptanib, and bevacizumab may disrupt retinal homeostasis due to the essential role of VEGF in retinal neurons and glial cells survival [6–8].

Müller glial cells constitute one of the VEGF sources in the retina, in addition to retinal pigment epithelium cells, astrocytes, and ganglion cells [9–11]. Müller cells play a crucial role in maintaining retinal homeostasis, structure and function [12]. In pathological conditions, Müller cells undergo reactive gliosis characterized by hypertrophy, increased intermediate filaments (e.g., glial fibrillary acidic protein and vimentin), proliferation, and production of inflammatory and angiogenic factors [13–15]. Cytokines and growth factors play a role in cell survival and defense, and Müller cells participate by producing molecules such as basic fibroblast growth factor (bFGF), glial cell line-derived neurotrophic factor, pigment epithelium-derived factor, tumor necrosis factor-alpha (TNF-α), interleukin-1 beta (IL-1β), IL-6 and, most importantly, VEGF [16].

Enhanced VEGF expression stimulates vascularization to supply the need for oxygen and nutrients during hypoxia [17], a condition occurring in vascular retinopathies due to cytokine upregulation and inflammatory cell influx, followed by vascular occlusion and ischemia. The relationship between hypoxia and inflammation is subject to controversy. For example, VEGF upregulation induced by hypoxia was not mediated by inflammation in a study using the human retinal pigment epithelial (RPE) cell line ARPE-19 [18].

In some cases, inflammation develops before hypoxia, as shown in primary human Müller cells, in which VEGF-A secretion has also been shown to be upregulated by IL-6 [19]. Other factors may account for VEGF secretion under inflammatory conditions, such as platelet-derived growth factor-BB (PDGF-BB), heparin-binding epidermal growth factor-like growth factor (EGF), and bFGF-increased VEGF content in MIO-M1 Müller cells [20].

On the other hand, in ARPE-19 cells VEGF inhibition can increase the levels of IL-8, which takes on the role as angiogenic agent in the absence of VEGF [21]. In another study, neither VEGF inhibition nor recombinant VEGF-A raised IL1-β, IL-6 and IL-8 levels in MIO-M1 cells, as observed for bFGF [22].

Considering that Müller cells are a potential source of VEGF [12] and that modulators can sometimes have combined effects, we evaluated the effect of aflibercept (AFL) and bevacizumab (BVZ) on cell viability/metabolism, and cytokine and growth factor production by Müller cells subjected to hypoxia *in vitro*. Understanding the behavior of retinal cells within the microenvironment, particularly under the influence of anti-VEGF agents, is crucial for enhancing follow-up protocols and exploring potential combinations with adjunctive drugs.

## Materials and methods

### Materials

Dulbecco Modified Eagle Medium (DMEM) high glucose, penicillin-streptomycin, and fetal bovine serum were obtained from Invitrogen (Life Technologies, Carlsbad, CA, USA). Aflibercept (AFL) was purchased from Bayer S/A (Eylea^®^ solution for injection, 40 mg/mL, São Paulo, Brazil). Bevacizumab (BVZ) was obtained from Produtos Roche and Químicos Farmacêuticos Ltda (Avastin^®^ solution for injection, 25 mg/mL, São Paulo, Brazil). Sodium bicarbonate and cobalt chloride were obtained from Sigma-Aldrich Quimica Ltda (São Paulo, Brazil). TNF-α, IL-1β, IL-6, IL-8, PDGF-BB, EGF, VEGF and bFGF levels were measured with a commercial kit (Luminex Human Magnetic Assay, R&D Systems). All other reagents used were of analytical grade.

#### Human Müller cell culture (MIO-M1)

The human Müller cell line MIO-M1 (Moorfields/Institute of Ophthalmology-Müller 1) [23] was obtained from Dr. G. A. Limb (UCL Institute of Ophthalmology, University of London, UK) through a license agreement with UCLB-XIP, London, UK. The cells were cultured in media containing DMEM high glucose supplemented with 10% fetal bovine serum, 1% penicillin streptomycin, and 44 mM sodium bicarbonate. For cell viability analyses, 2x10^5^ cells/well were grown on 6 well plates (growth area 9.5 cm^2^/well) for Trypan blue dye exclusion test and 2.5x10^4^ cells/well were grown on 96 well plates (growth area 0.32 cm^2^/well) for MTT assay (Corning® Inc., NY, USA). For analyte detection, 2 x 10^5^ cells/well were grown on 6 well plates: (growth area 9.5 cm^2^/well) (Corning^®^ Inc., NY, USA). All cells were maintained for 24 h for adherence, followed by incubation for 12 h in serum-free culture medium for synchronization in the nonproliferative state, prior to hypoxia and anti-VEGF treatments. Cobalt chloride was used as a hypoxia-mimetic agent [24]. The cells were then subjected to the following treatments for 24, 48, or 72 hours: a) culture media alone; b) 400 μM cobalt chloride diluted in culture media to mimic hypoxia; c) anti-VEGF drugs (AFL or BVZ) diluted in culture media at a concentration of 0.5 mg/mL, corresponding to clinical doses; or d) 400 μM cobalt chloride + anti-VEGF drugs (AFL or BVZ) at 0.5 mg/mL diluted in culture media. The concentrations of anti-VEGF drugs were estimated by dividing the commonly used dose of AFL or BVZ for the treatment of retinal disease in humans (2 mg per eye) by the volume of the eye. The concentration used for both drugs was 0.5 mg/mL.

### Methods

#### Cell viability assays

*Trypan blue dye exclusion test*. To determine the number of dead (blue staining) and live (clear cytoplasm) cells, MIO-M1 cells were transferred to a 6 well plate for adherence during 24 h and then incubated at serum-free culture medium for 12 h. The cells were submitted to the treatments mentioned above (control groups were composed of cells maintained with culture medium with no treatments). Samples were also obtained to detected cell viability before the periods of incubation (0 h). The cells were subsequently detached from the plate surface using a 0.5% trypsin-EDTA solution (Sigma Aldrich) for 3 min at 37° C. Fetal bovine serum (v: v) (Gibco by Life Technologies) was added to inhibit trypsin activity. The supernatant was centrifuged, and the pellet was diluted in fresh media. A 10-μL aliquot of MIO-M1 cell suspension was diluted in 0.4% trypan blue stain (v: v) (Gibco by Life Technologies) and counted in an automated cell counter (Countess® Automated Cell Counter, Invitrogen). Cell viability was expressed as percentage of control 0 h. At least six replicates were performed for each condition.

*MTT assay*. Following the same previous procedure, MIO-M1 cells were cultured on 96 well plates and incubated according to the treatments and times mentioned previously. The MTT assay is a colorimetric assay used to measure mitochondrial activity, i.e., cell metabolism, and indirectly estimates the number of viable cells. As previously described [25], a 10-μL MTT solution diluted in 0.1M PBS (5 mg/mL) was added to each well, and the cells were incubated at 37°C for 4 hours. After discarded the media, 100 μL DMSO was added to each well and the plate was placed under agitation at room temperature for 30 min to dissolve intracellular MTT formazan crystals. Cell viability/metabolism was expressed as optical density. The optical density was measured at 570 nm with a reference wavelength of 690 nm (Spectra Max 190, Molecular Devices; San Francisco, CA). At least six replicates were performed for each condition.

#### Cytokine and growth factor multiplex analysis

Cell culture medium supernatant was placed in microtubes and immediately frozen at -80⁰C for posterior analysis. A mixture of TNF-α, IL-1β, IL-6, IL-8, PDGF-BB, EGF, VEGF and FGF-2 antibody beads was added to a 96-well filter plate containing standards and culture medium for cell treatment. The culture medium was previously diluted 10-fold in assay buffer to achieve a final volume of 25 μL. Analyte measurements were performed according to the manufacturers’ instructions. A Bioplex^®^ 200 System (Bio-Rad, Hercules, California, USA) was used to quantify the bead-specific R-PE fluorescence. Three replicates were performed for each condition.

#### Statistical analysis

The results were expressed as mean ± standard deviation (SD). Differences between conditions (untreated, hypoxia, anti-VEGF treatment, hypoxia + anti-VEGF treatment) in each exposure time (24 h, 48 h and 72 h) were analyzed with two-way ANOVA, followed by Bonferroni’s *post hoc* test. The level of statistical relevance was set at 5% (*p*<0.05).

## Results

**Fig 1:**
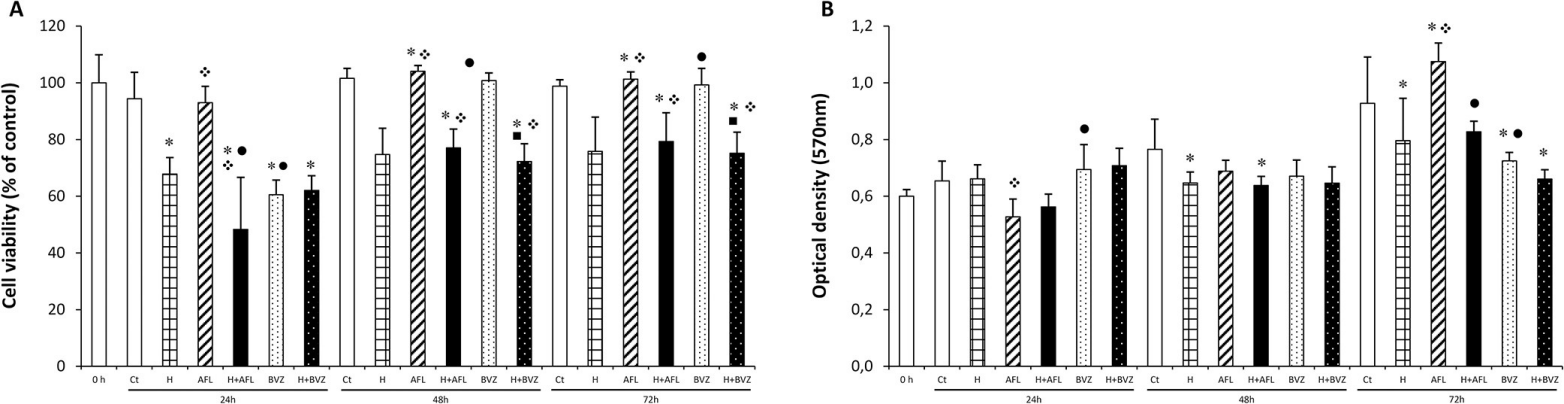
Effect of treatment with aflibercept (AFL; 0.5 mg/mL), hypoxia (H; 400 μM CoCl_2_), hypoxia + aflibercept (H+AFL), bevacizumab (BVZ; 0.5 mg/mL) and hypoxia + bevacizumab (H + BVZ) on cell viability by Trypan Blue (A) and MTT assays (B). Trypan blue results are expressed as percentage to the control 0 h. The control (0 h) are untreated MIO-M1 cells kept in serum-free culture medium for 12 h before treatment. MTT assay values are expressed according to the optical density at 570 nm of wavelength. The results are expressed as mean ± standard deviation for bar charts based on for each treatment. two-way ANOVA and Bonferroni *post hoc p*<0.05. *p<0.05 vs control; ❖*p*<0.05 vs hypoxia; ●*p*<0.05 vs AFL; ◼*p*<0.05 vs BVZ.

The effects of AFL and BVZ on cell viability and metabolism of MIO-M1 Müller cells under normoxia and hypoxia were investigated by using trypan blue and MTT assays, respectively (Fig 1).

In the trypan blue assay, AFL increased cell viability at 48 h (*p*<0.05) and 72 h (*p*<0.05) when compared to their respective controls. In contrast, there was a significant decrease in cell viability in BVZ-treated cells at 24 h (*p*<0.05) compared to controls but not in other treatment times. BVZ significantly reduced cell viability at all times compared to AFL treatment. (Fig 1A).

Hypoxia led to a decreased cell viability, but it was statistically significant only at 24 h (at 24 h: hypoxia *vs* control, *p*<0.05). In hypoxic cells treated with AFL, the combined conditions intensified the decrease of cell viability compared to AFL or hypoxia alone (*p*<0.05) at 24 h. In contrast, at 48 h and 72 h, the combined conditions increased cell viability compared to hypoxia treated cells (*p*<0.05). In cells treated with both BVZ and hypoxia, there was a reduced cell viability compared to BVZ or hypoxia alone (*p*<0.05) at 48 h and 72 h, but not at 24 h (Fig 1A).

In MTT assay, AFL marginally increased the cell viability/metabolism of MIO-M1 cells at 72 h (*p* = 0.054), but it had no significant effect at 24 h or 48 h compared to their respective controls. On the other hand, BVZ decreased metabolism at 72 h (*p*<0.05), while there was no difference between BVZ and control at other exposure times.

Hypoxia resulted in a decrease in cell metabolism at 48 h (p = 0.05) and 72 h (*p*<0.05) compared to their respective controls, while it had no significant effect at 24 h (Fig 1B).

Regarding the anti-VEGF treatments in cells under hypoxia, the combined hypoxia and AFL treatment led to a decreased metabolism only at 72 h compared to AFL treatment alone (*p*<0.05). There were no difference between hypoxia and AFL treatment compared to hypoxia or AFL alone at 24h or 48 h. There was no difference between combined hypoxia and BVZ treatment and hypoxia or BVZ alone at any exposure time (Fig 1B).

### Growth factor and cytokines

**Fig 2:**
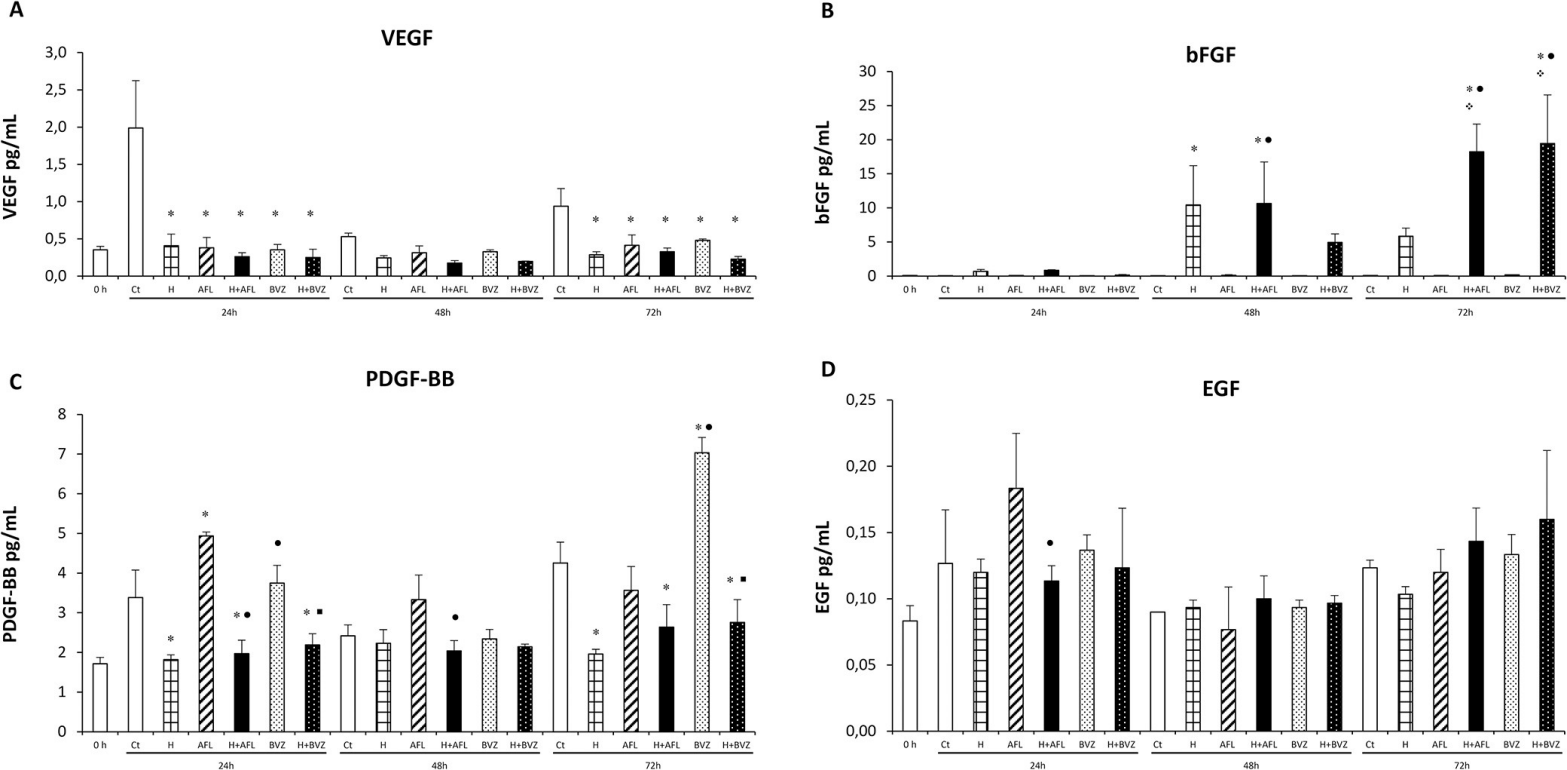
Effect of treatment with aflibercept (AFL; 0.5 mg/mL), hypoxia (H; 400 μM CoCl_2_), hypoxia + aflibercept (H+AFL), bevacizumab (BVZ; 0.5 mg/mL) and hypoxia + bevacizumab (H + BVZ) on VEGF (A), bFGF (B), PDGF-BB (C) and EGF (D) levels in the supernatant of MIO-M1 cells at 24 h, 48 h and 72 h. The 0 h time corresponds to baseline. The control group (0 h) consisted of untreated MIO-M1 cells maintained in serum-free culture medium for 12 h prior to treatment. The results are expressed as mean ± standard deviation for bar charts based on triplicates for each treatment. two-way ANOVA and Bonferroni *post hoc p*<0.05. *p<0.05 vs control; ❖*p*<0.05 vs hypoxia; ●*p*<0.05 vs AFL; ◼*p*<0.05 vs BVZ.

**Fig 3:**
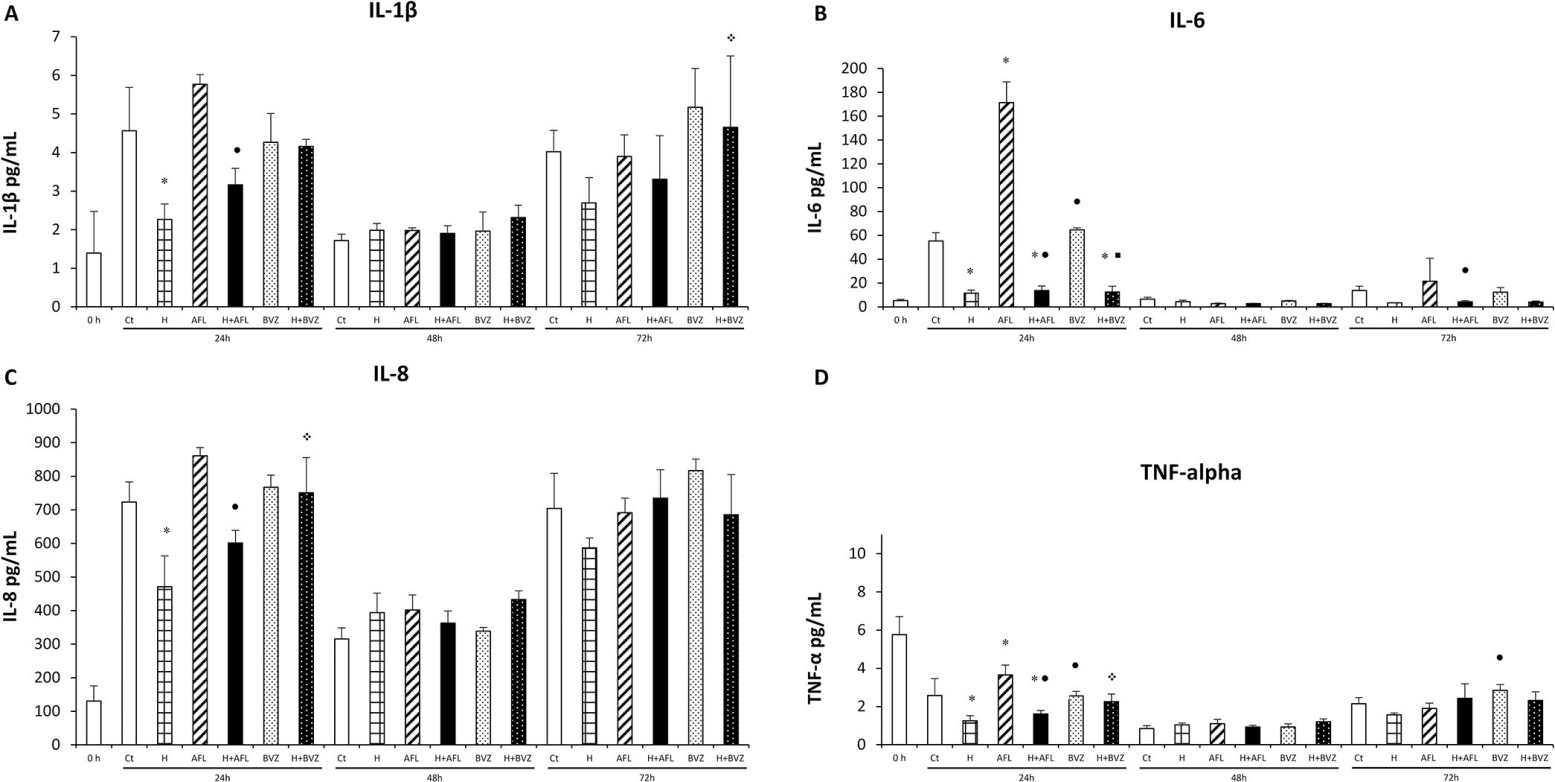
Effect of treatment with aflibercept (AFL; 0.5 mg/mL), hypoxia (H; 400 μM CoCl_2_), hypoxia + aflibercept (H+AFL), bevacizumab (BVZ; 0.5 mg/mL) and hypoxia + bevacizumab (H + BVZ) on IL-1β (A), IL-6 (B), IL-8 (C) and TNF-α (D) levels in the supernatant of MIO-M1 cells at 24 h, 48 h and 72 h. The 0 h time corresponds to baseline. The control group (0 h) consisted of untreated MIO-M1 cells maintained in serum-free culture medium for 12 h prior to treatment. The results are expressed as mean ± standard deviation for bar charts based on triplicates for each treatment. Two-way ANOVA and Bonferroni *post hoc* at the level of *p*<0.05. *p<0.05 vs control; ❖*p*<0.05 vs hypoxia; ●*p*<0.05 vs AFL; ◼*p*<0.05 vs BVZ.

Figs 2 and 3 show the expression levels of the growth factors VEGF, bFGF, PDGF-BB and EGF, as well as the cytokines IL-1β, Il-6, IL-8 and TNF-α produced by MIO-M1 cells in response to the treatment.

#### At 24 h

At 24 h, VEGF levels treated with AFL and BVZ were until 8 times lower (*p*<0.05; Fig 2), regardless of exposure to cobalt chloride compared to controls. Hypoxia also reduced VEGF levels at 24h (*p*<0.05).

Basic FGF were statistically similar for all anti-VEGF treatments and control.

PDGF-BB significantly increased with the anti-VEGFs in relation to control (*p*<0.05), but decreased with hypoxia treatment, regardless of anti-VEGF treatments (*p*<0.05).

No significant differences were observed between the treatments (AFL or BVZ) or hypoxia alone and the controls with respect to EGF levels. The EGF-reducing effect of hypoxia was only significant in cells treated with AFL compared to AFL treatment alone (*p*<0.05). Treatment with BVZ alone and H+BVZ yielded similar results.

Regarding the cytokines (Fig 3), no differences in IL-1β levels were observed between cells treated with anti-VEGFs and control. In contrast, hypoxia significantly reduced IL-1β levels at 24 h (*p*<0.05). A comparison between AFL alone and H+AFL shows that the effect of hypoxia was stronger than the effect of AFL, resulting in lower IL-1β levels in MIO-M1 cells (*p*<0.05). In contrast, treatment with BVZ alone relative to H+BVZ shows that the effect of BVZ was stronger than the effect of hypoxia, resulting in a marginally significant increase in IL-1β levels in BVZ alone (*p* = 0.055).

IL-6 levels was nearly 3-fold increase in cells exposed to AFL, but not to BVZ, relative to control (*p*<0.05). Hypoxia significantly decreased IL-6 levels in MIO-M1 cells relative to control (*p*<0.05). Comparing H+AFL to AFL alone and its respective control showed that AFL was unable to inhibit the effect of hypoxia, maintaining the decreased of IL-6 levels at this time of exposure (*p*<0.05). The effect of hypoxia was also stronger than the effect of BVZ (BVZ *vs* H+BVZ and Ct *vs* H + BVZ), resulting in reduced IL-6 levels (*p*<0.05). BVZ treatment showed lower IL-6 levels than AFL at 24 h (*p*<0.05).

Treatment with AFL or BVZ alone had no effect on IL-8 levels relative to control. However, hypoxia significantly decreased IL-8 levels in MIO-M1 cells compared to untreated cells (*p*<0.05), where the presence of BVZ prevented hypoxia from reducing IL-8 levels in MIO-M1 cells, but not AFL.

For TNF-α, treatment with AFL significantly increased TNF-α levels compared to control at 24 h (*p*<0.05), but BVZ had no effect. Hypoxia reduced TNF-α levels significantly in relation to control at 24 h (*p*<0.05). In cells under hypoxia, the effect of hypoxia was stronger than the effect of AFL, resulting in decreased TNF-α levels in H+AFL when compared to AFL alone (*p*<0.05). When comparing H+BVZ to hypoxia, BVZ in hypoxic cells maintained higher TNF-α levels at this time of exposure (*p*<0.05). BVZ treatment showed lower TNF-α levels compared to AFL alone (*p*<0.05).

#### At 48 h

At 48 h, VEGF and EGF levels did not differ among the groups (Fig 2).

For bFGF, AFL and BVZ treatments had similar bFGF levels compared to the control, but were significantly higher in cells exposed to hypoxia. AFL treatment combined with hypoxia had higher levels at 48 h compared to the control and AFL alone (*p*<0.05).

Treatments and controls yielded similar results for PDGF-BB levels. Also, no difference was observed at hypoxia alone.

No differences in IL-1β, IL-6, IL-8 or TNF-α levels were observed among the control and experimental groups at 48 h (Fig 3).

#### At 72 h

VEGF levels in the supernatant of normoxic and hypoxic MIO-M1 cells treated with AFL and BVZ were 4 times lower compared to control at 72 h (*p*<0.05; Fig 2). Hypoxia alone also reduced VEGF levels at this time of exposure (*p*<0.05).

Basic FGF levels were statistically similar for all anti-VEGF treatments and control, but were significantly higher with hypoxia treatment (*p*<0.05). Also, anti-VEGF treatments combined with hypoxia yielded increased bFGF levels when compared to their respective controls, hypoxia or anti-VEGF treatments alone (*p*<0.05 for all comparisons).

Treatment with BVZ, but not with AFL, significantly increased PDGF-BB levels in relation to control (*p*<0.05). Hypoxia alone significantly reduced PDGF-BB levels. Hypoxia combined with BVZ and AFL also reduced PDGF-BB levels at 72 h (*p*<0.05 as shown by comparing to their respective controls and anti-VEGFs alone, except for the comparison between AFL combined to hypoxia and AFL alone).

At this time of exposure, no significant differences were observed between the treatments (AFL or BVZ) in normoxic and hypoxic cells and the control with respect to EGF levels.

In relation to the cytokines (Fig 3), no differences in IL-1β levels were observed between cells treated with anti-VEGFs or hypoxia and the control at 72 h. Treatment with BVZ alone relative to H+BVZ shows that the effect of BVZ was stronger than the effect of hypoxia, resulting in high IL-1β levels in MIO-M1 cells (*p*<0.05).

For IL-6 levels, no difference was observed when comparing AFL, BVZ or hypoxia to the control. Comparing H+AFL to AFL alone showed that AFL was unable to inhibit the effect of hypoxia, maintaining the decreased of IL-6 levels (*p*<0.05).

No differences in IL-8 were observed among the groups control and experimental at 72 h.

Also, no differences were observed among the groups for TNF-α levels, except for the comparison between the two anti-VEGFs, in which showed higher TNF-α levels with BVZ treatment (*p*<0.05).

## Discussion

In this study, we investigated the effects of AFL and BVZ on cell viability and metabolism, as well as on several growth factors (VEGF, bFGF, PDGF-BB, and EGF) and cytokines (IL-1β, IL-6, IL-8 and TNF-α) in MIO-M1 Müller cells submitted to hypoxia. Since the cellular response varies according to the microenvironment, we mimic the hypoxic conditions observed in vascular retinopathies, such as those encountered in diabetic patients [26].

The choice of bevacizumab was based on the fact that in a previous study, we had already investigated the effect of this anti-VEGF in a shorter exposure time [27]. In this study, our proposal was to extend the exposure time of bevacizumab to Müller cells. There is also the consideration of its cost-effectiveness, making it widely used for the treatment of vascular retinopathies in clinics and outpatient settings over the world [28, 29]. Our choice of aflibercept is justified by studies indicating its greater availability in the macula and its ability to bind to multiple growth factors [30].

In our study, AFL did not show difference in cell viability in short term treatment (24h). Indeed, the studies evaluating MIO-M1 cell viability *in vitro* did not find changes with AFL [30–32]. Concerning cell metabolism, some authors showed a decrease in relative cell growth with the MTT assay [30]. Although not statistically significant, we observed a tendency to decrease cell viability/metabolism with AFL in short term exposure. In longer treatments, increased cell viability and metabolism with AFL could be a response of Müller cells indicative of gliosis. This is the first study evaluating the long-term effect of AFL in human Müller cells. Furthermore, as we know, no previous studies evaluated the prolonged effect of this anti-VEGF on these cells.

In contrast, BVZ decreased cell viability and did not alter cell metabolism at the beginning of the treatment, but significantly decreased cell metabolism in the long-term. Although previous studies did not report changes in cell viability assays of MIO-M1 cells treated with BVZ in short-term exposure [30], an increase in apoptosis, indicative of cell death, was observed.

According to our data, cell viability and metabolism in MIO-M1 cells can be affected by hypoxia and BVZ. In turn, AFL probably acts in these cells inducing gliosis. These differences in cell viability and metabolism between the anti-VEGFs could be explained by BVZ effects on mitochondrial toxicity. A greater reduction in mitochondrial membrane potential was observed for BVZ-treated ARPE-19 cells in culture [33]. However, any extrapolation in MIO-M1 cells must be done with caution since there are other retinal cells in the microenvironment that respond to the intravitreal injection with anti-VEGFs.

Also, hypoxia reduced Müller cell viability and metabolism. Previous studies have already described an increase in cell death in cultured rat Müller cell line (rMC-1) after 24 h treatment with cobalt chloride [34]. Similarly, a loss of cell viability in MIO-M1 cells treated with cobalt chloride in different concentrations, including the same one used in our study (400 μM), has been documented [35, 36].

When anti-VEGFs were added to the hypoxic cells, the effect of hypoxia in cell viability prevailed over the effect of anti-VEGFs, i.e., decreased cell viability in cells under hypoxia did not change with AFL or BVZ treatments or the cell metabolism with AFL. It means that anti-VEGF treatments could not reverse the loss of Müller cell viability or repair cell metabolism in a hypoxic situation of our study. Indeed, there was a loss of ganglion cells [37] and Müller cells [38] during hypoxia in retinopathies treated with anti-VEGFs. Treatment with AFL also failed to recover the loss of cell viability in hypoxic Müller cells [31]. A recent study showed that an initial dose of intravitreal BVZ was associated with impairment in foveal Müller cell cone structure and poor visual acuity after one month of treatment in diabetic macular edema (DME) patients [39].

Since Müller cells are involved in various functions in the retina, such as neurotransmission, ion and nutrients regulation, metabolites recycling, and blood barrier contribution, any disturbance to Müller cells indeed affects their functions and, consequently, the maintenance of neurons in the retina. Loss of Müller cell viability, mainly their endfeet, causes the breakdown of the blood-retinal barrier and increased vascular permeability [40]. Based on the literature and in our findings, an altered hypoxic microenvironment may affect Müller cell viability and metabolism, as well as impair its function in the retina, affecting the neuronal support. It seems that anti-VEGF drugs cannot repair these cell alterations.

As expected, clinical doses of BVZ and AFL reduced VEGF levels in MIO-M1 cells, matching the results of other studies [27, 30]. Both compounds are humanized monoclonal antibodies binding to circulating VEGF and preventing it from binding to its cellular receptor [41]. Indeed, previous studies have shown that BVZ and AFL intravitreal applications decreased VEGF levels [42–45]. In patients with choroidal neovascularization from age-related macular degeneration (AMD), vitreous VEGF levels were ~ 10 times lower in the first and second month of follow-up after treatment with 1.25 mg BVZ [42]. Likewise, AFL reduced VEGF levels about 2–3 times after 2 and 4 months of treatment for DME [44]. VEGF was undetectable at five months of treatment with two AFL applications in patients with and without macular atrophy [46].

In the present study, the selection of the 400 μM CoCl_2_ concentration for our experiments was based on the findings of Kim et al., who performed a series of experiments with different CoCl_2_ concentrations and observed the highest level of hypoxia at 400 μM CoCl_2_ in MIO-M1 cells [35].

In hypoxia, although most studies found an increase in VEGF levels in MIO-M1 cells [47–49],_msocom_1 we observed a decrease of its levels. A possible explanation for that may be the 25mM glucose normally used for growth and maintenance of MIO-M1 cells in culture. Together with hypoxia, a 25mM glucose concentration is associated with a reduction of VEGF levels in MIO-M1 cells [50], in cells from diabetic patients, and in ischemic tissue from diabetic animals [51]. A decrease in plasma VEGF levels was also observed in healthy subjects with hyperglycemia intervention [52]. Also, different induction methods to mimic hypoxia in MIO-M1 cells, such as low oxygen using chambers [47] and different exposure times [48], as well as CoC_l2_ concentrations [49], may result in different VEGF levels. Although low levels of VEGF help to inhibit vascularization in vascular retinopathies, VEGF plays a protective role in the survival of endothelial cells and retinal neurons [6, 7, 12]. Then, decreasing VEGF production could lead to greater susceptibility to stressors and, consequently, impairment in cell viability.

Besides VEGF, TNF-α, IL-1β, IL-6, IL-8, and PDGF-BB decreased in cells submitted to hypoxia in short-term treatment. Like VEGF, hypoxia can interfere with immune response, leaving the cells susceptible to stressors. Hypoxia generates an immunosuppressive environment and, together with an elevated glycolytic activity, increases lactate, protons, and carbonic acid in the extracellular media, generating acidosis that contributes to immunosuppression and impairment in cytokine production [53, 54].

Contrary to cytokines and growth factors cited above, we observed that bFGF markedly increased in hypoxic conditions. bFGF produced by Müller cells also acts as an angiogenic agent without VEGF [55], and co-localized with glial fibrillary acid protein (GFAP), an indicator of gliosis, in a fibrovascular membrane of a patient with proliferative diabetic retinopathy [56]. Moreover, bFGF stimulates the survival, proliferation, migration, and differentiation of endothelial cells [57] and Müller cells [55] and was elevated in the retina of patients affected by diabetic retinopathy [58, 59]; however, some authors found no pre- and post-treatment differences [60]. In our study, bFGF levels remained unchanged when treated with anti-VEGFs in normoxic cells but increased under hypoxia and were intensified by the presence of anti-VEGFs. Similar to the present study, Yafai et al. observed that bFGF levels increased in proportion to the duration of hypoxia [56]. Then, VEGF downregulation induced by hypoxia or anti-VEGF allows us to infer that increased bFGF production could be one of the compensatory mechanisms by the VEGF downregulation.

Another evaluated growth factor, EGF, is reportedly mitogenic for Müller glial cells and increases in response to retinal damage [61]. Malik et al. detected increased EGF levels after intravitreal BVZ injection in the vitreous of DME patients [62]. However, we did not observe increased EGF levels with hypoxia or anti-VEGF treatments.

PDGF-BB is also involved in choroidal neovascularization, contributing to the neovascularization and retinal detachment induced by VEGF [63]. However, in our study, PDGF-BB seems to act VEGF-independent, since both AFL and BVZ enhanced PDGF-BB levels at 24 h and 72 h, respectively. In fact, like bFGF, PDGF-BB is an angiogenic agent, stimulating neovascularization and leading to treatment resistance [64]. It explains the increased levels of PDGF-BB in MIO-M1 cells following exposure to AFL and BVZ. In contrast, hypoxia decreased PDGF-BB levels, but anti-VEGF treatments in hypoxic cells did not restore basal PDGF-BB levels.

Regarding the cytokines, anti-VEGFs did not impact IL-1β levels in normoxic cells but increased with BVZ treatment in hypoxic cells in the short and long term. Rezzola et al. did not find changes in the IL-1β levels of MIO-M1 cell mRNA following treatment with ranibizumab [22]. High levels of this cytokine were associated with cone segment degeneration, induction of inflammasome components (e.g., IL-6), and macrophage recruitment [65, 66]. Based on our findings, in general BVZ, but not AFL, elicited inflammation in cells under hypoxia.

IL-6 levels were markedly higher after treatment with AFL in MIO-M1 cells under normoxia at 24 h, but maintained low in cells under hypoxia, suggesting that AFL treatment cannot revert the IL-6 decrease induced by hypoxia in MIO-M1 cells in our study conditions. An increase in IL-6 levels in aqueous humor were detected one day after intravitreous BVZ administration for diabetic retinopathy [67], and 10 days after treatment for proliferative diabetic retinopathy [68]. Upregulation of IL-6 levels have been associated with developing resistance to BVZ in AMD [69]. It means that anti-VEGFs can induce acute inflammation in the retina microenvironment.

We did not detect differences in IL-8 levels in cells treated with BVZ and AFL alone compared to controls, but BVZ increased IL-8 levels in cells under hypoxia in short term treatment. Indeed, the studies are controversial, as observed for AFL treatments that increased IL-8 levels [45] or not [31, 46]. In AMD and choroidal neovascularization, IL-8 levels were ~300 times higher in the second month of follow-up than at baseline. On the other hand, no pre/post-treatment differences in IL-8 levels were observed in DME treated with 1.5 mg BVZ [70]. Recently, patients with DME receiving anti-VEGF agents had high IL-8 levels in the aqueous humor, with no positive change in macula thickness [71]. IL-8 may be part of a compensatory mechanism for angiogenic stimulation in response to VEGF inhibition, as observed in our study with BVZ treatment in hypoxic cells and in the study of Cabral et al. [42].

Regarding TNF-α, we observed increased levels when cells were treated with AFL under normoxia and BVZ under hypoxia. In hypoxic cells, AFL showed lower levels of this cytokine compared to controls or AFL alone, showing the predominance of hypoxia over AFL effect. Lazzara et al. reported a decrease in TNF-α after AFL treatment in non-proliferative diabetic retinopathy [72]. Abcouwer et al. did not show changes in the TNF-α mRNA after BVZ intravitreal injection in rat model ischemic retinal disease [73]. In addition to being a growth factor, TNF-α is an angiogenic and inflammatory molecule involved in choroidal neovascularization and AMD. In a study comparing intravitreal administration of TNF-α inhibitors to anti-VEGF treatment, TNF-α inhibitors suppressed the expression of pro-angiogenic factors and reduced vascular tube formation and retinal neovascularization [74]. Also, monoclonal anti-TNFα antibodies prevented retinal degeneration in a murine model of retinitis pigmentosa [75]. Like our findings for IL-1β, BVZ induces inflammation in cells under hypoxia.

Blue light exposure induces oxidative stress, such as hypoxia, and aggravates the progression of age-related macular degeneration. Matching some of our results, Sato et al. observed that blue light irradiation decreased IL-6, IL-7, IL-8, MCP-1, and VEGF levels and increased bFGF levels in retinal pigment epithelium cells *in vitro***.** However, AFL and ranibizumab, with or without exposure to blue light, reduced the levels of IL-6, IL-8, and bFGF [76].

A limitation to our study is the presence of high glucose in the media used for MIO-M1 cell maintenance, which in combination with hypoxia could potentially interfere in cytokine and growth factors response. Furthermore, CoCl_2_ as a hypoxia inductor has some criticisms as it influences the transcription of different genes that were not affected by the low oxygen-induced hypoxia chamber, produces oxidative stress through ROS generation and can regulate other genes independently of HIFs [77]. Moreover, although the MIO-M1 cell lineage is a widely employed Müller cell model, *in vitro* conditions may not fully replicate the complex clinical conditions and compensatory actions taking place simultaneously *in vivo*. However, the study of isolated Müller cells under the effects of anti-VEGFs help us to understand the role of these cells in retinal maintenance and treatment resistance. This is a translational study that can help to understand the molecular-level effects of anti-VEGFs, enabling the improvement of existing drugs and therapeutic combinations that trigger minimal side effects in the treatment of patients with vascular retinopathies.

## Conclusions

In our study, CoCl_2_-induced hypoxia and BVZ affected MIO-M1 cell viability and metabolism, whereas AFL probably induced gliosis. Hypoxia induced acute immunosuppression and based on bFGF levels, probably induces fibrosis in Müller cells. BVZ induced acute inflammation in hypoxic cells, but AFL did not affect cytokine and growth factor levels in cells under hypoxia.

It is reasonable to assume that cell viability and metabolism are affected in diabetic retinal diseases by changes in the microenvironment, and anti-VEGFs treatments can intensify these changes since VEGF and other factors are essential to cell survival and maintenance. Also, hypoxia and anti-VEGFs induced inflammation and gliosis by the expression of other molecules to compensate the microenvironment altered by the disease. These findings highlight the need for developing new adjuvants for anti-VEGF treatment capable of preserving the retina.

## Supporting information

S1 Table Trypan blue data of the control and experimental groups at 24 h, 48 h and 72 h. (PDF)

S2 Table MTT data of the control and experimental groups at 24 h, 48 h and 72 h. (PDF)

S3 Table Cytokine data of the control and experimental groups at 24 h, 48 h and 72 h. (PDF)
