# Supplementary material for: Effects of aflibercept and bevacizumab on cell viability, cell metabolism and inflammation in hypoxic human Müller cells
Source: PLoS One. 2024 Mar 27;19(3):e0300370. doi: 10.1371/journal.pone.0300370 (PMC10971667; doi:10.1371/journal.pone.0300370)
Supplement: S1 Table — (PDF) [file pone.0300370.s001.pdf]

**Group**	**TrypanBlue (%)**
Ct 0h	70
Ct 0h	102
Ct 0h	94
Ct 0h	96
Ct 0h	106
Ct 0h	104
Ct 0h	106
Ct 0h	103
Ct 0h	98
Ct 0h	104
Ct 0h	106
Ct 0h	102
Ct 0h	106
Ct 24h	85
Ct 24h	106
Ct 24h	96
Ct 24h	101
Ct 24h	96
Ct 24h	82
H 24h	71
H 24h	65
H 24h	79
H 24h	63
H 24h	64
H 24h	65
H 24h	71
H 24h	65
H 24h	79
H 24h	63
H 24h	64
AFL 24h	88
AFL 24h	88
AFL 24h	99
AFL 24h	100
AFL 24h	87
AFL 24h	95
H+AFL 24h	31
H+AFL 24h	50
H+AFL 24h	73
H+AFL 24h	23
H+AFL 24h	53
H+AFL 24h	59
BVZ 24h	53
BVZ 24h	56
BVZ 24h	66
BVZ 24h	65
BVZ 24h	59
BVZ 24h	64

H+BVZ 24h	62
H+BVZ 24h	63
H+BVZ 24h	66
H+BVZ 24h	55
H+BVZ 24h	59
H+BVZ 24h	53
H+BVZ 24h	65
H+BVZ 24h	69
H+BVZ 24h	67
H+BVZ 24h	62
Ct 48h	103
Ct 48h	101
Ct 48h	102
Ct 48h	103
Ct 48h	101
Ct 48h	102
Ct 48h	95
Ct 48h	102
Ct 48h	106
Ct 48h	103
Ct 48h	95
Ct 48h	104
H 48h	67
H 48h	87
H 48h	75
H 48h	80
H 48h	64
H 48h	63
H 48h	59
H 48h	65
H 48h	78
H 48h	78
H 48h	85
H 48h	76
H 48h	79
H 48h	87
H 48h	78
H 48h	79
H 48h	88
H 48h	80
H 48h	62
H 48h	67
AFL 48h	104
AFL 48h	103
AFL 48h	103
AFL 48h	105
AFL 48h	101
AFL 48h	103
AFL 48h	104

AFL 48h	106
AFL 48h	104
AFL 48h	100
AFL 48h	106
AFL 48h	106
H+AFL 48h	82
H+AFL 48h	66
H+AFL 48h	67
H+AFL 48h	67
H+AFL 48h	76
H+AFL 48h	64
H+AFL 48h	79
H+AFL 48h	73
H+AFL 48h	61
H+AFL 48h	79
H+AFL 48h	72
H+AFL 48h	72
H+AFL 48h	79
H+AFL 48h	92
H+AFL 48h	92
H+AFL 48h	73
H+AFL 48h	79
H+AFL 48h	83
H+AFL 48h	95
H+AFL 48h	90
BVZ 48h	98
BVZ 48h	99
BVZ 48h	100
BVZ 48h	102
BVZ 48h	105
BVZ 48h	100
H+BVZ 48h	69
H+BVZ 48h	66
H+BVZ 48h	78
H+BVZ 48h	75
H+BVZ 48h	69
H+BVZ 48h	63
H+BVZ 48h	65
H+BVZ 48h	80
H+BVZ 48h	75
H+BVZ 48h	71
H+BVZ 48h	72
H+BVZ 48h	84
Ct 72h	99
Ct 72h	100
Ct 72h	98
Ct 72h	100
Ct 72h	101
Ct 72h	97

Ct 72h	99
Ct 72h	98
Ct 72h	102
Ct 72h	100
Ct 72h	94
Ct 72h	98
H 72h	56
H 72h	60
H 72h	68
H 72h	73
H 72h	71
H 72h	68
H 72h	71
H 72h	67
H 72h	78
H 72h	73
H 72h	78
H 72h	80
H 72h	106
H 72h	85
H 72h	88
H 72h	87
H 72h	79
H 72h	75
AFL 72h	103
AFL 72h	101
AFL 72h	102
AFL 72h	103
AFL 72h	101
AFL 72h	102
AFL 72h	100
AFL 72h	102
AFL 72h	99
AFL 72h	104
AFL 72h	95
AFL 72h	102
H+AFL 72h	71
H+AFL 72h	83
H+AFL 72h	83
H+AFL 72h	68
H+AFL 72h	87
H+AFL 72h	79
H+AFL 72h	83
H+AFL 72h	75
H+AFL 72h	78
H+AFL 72h	99
H+AFL 72h	88
H+AFL 72h	79
H+AFL 72h	59

H+AFL 72h	66
H+AFL 72h	88
H+AFL 72h	86
H+AFL 72h	80
H+AFL 72h	76
BVZ 72h	94
BVZ 72h	96
BVZ 72h	104
BVZ 72h	94
BVZ 72h	94
BVZ 72h	100
BVZ 72h	106
BVZ 72h	106
H+BVZ 72h	77
H+BVZ 72h	85
H+BVZ 72h	88
H+BVZ 72h	79
H+BVZ 72h	67
H+BVZ 72h	69
H+BVZ 72h	67
H+BVZ 72h	78
H+BVZ 72h	69
H+BVZ 72h	72
H+BVZ 72h	83
H+BVZ 72h	66
H+BVZ 72h	77

*Ct=control; AFL=aflibercept; H=hypoxia;
H+AFL=hypoxia+aflibercept;
BVZ=bevacizumab;
H+BVZ=hypoxia+bevacizumab
