# Supplementary material for: Effects of aflibercept and bevacizumab on cell viability, cell metabolism and inflammation in hypoxic human Müller cells
Source: PLoS One. 2024 Mar 27;19(3):e0300370. doi: 10.1371/journal.pone.0300370 (PMC10971667; doi:10.1371/journal.pone.0300370)
Supplement: S2 Table — (PDF) [file pone.0300370.s002.pdf]

**Group**	**MTT assay (OD)**
Ct 0h	0.61
Ct 0h	0.63
Ct 0h	0.57
Ct 0h	0.58
Ct 0h	0.58
Ct 0h	0.64
Ct 0h	0.63
Ct 0h	0.59
Ct 0h	0.59
Ct 0h	0.59
Ct 0h	0.59
Ct 24h	0.66
Ct 24h	0.63
Ct 24h	0.77
Ct 24h	0.75
Ct 24h	0.49
Ct 24h	0.55
Ct 24h	0.59
Ct 24h	0.63
Ct 24h	0.67
Ct 24h	0.64
H 24h	0.66
H 24h	0.62
H 24h	0.72
H 24h	0.73
H 24h	0.74
H 24h	0.65
H 24h	0.63
H 24h	0.62
H 24h	0.63
H 24h	0.61
AFL 24h	0.57
AFL 24h	0.56
AFL 24h	0.51
AFL 24h	0.58
AFL 24h	0.43
H+AFL 24h	0.63
H+AFL 24h	0.56
H+AFL 24h	0.55
H+AFL 24h	0.5
H+AFL 24h	0.57
BVZ 24h	0.63
BVZ 24h	0.61
BVZ 24h	0.71
BVZ 24h	0.69
BVZ 24h	0.83
H+BVZ 24h	0.66
H+BVZ 24h	0.64

H+BVZ 24h	0.74
H+BVZ 24h	0.71
H+BVZ 24h	0.79
Ct 48h	0.64
Ct 48h	0.7
Ct 48h	0.83
Ct 48h	0.91
Ct 48h	0.72
Ct 48h	0.71
Ct 48h	0.7
Ct 48h	0.97
Ct 48h	0.7
Ct 48h	0.79
H 48h	0.6
H 48h	0.57
H 48h	0.65
H 48h	0.66
H 48h	0.66
H 48h	0.64
H 48h	0.64
H 48h	0.7
H 48h	0.69
H 48h	0.65
AFL 48h	0.75
AFL 48h	0.68
AFL 48h	0.67
AFL 48h	0.71
AFL 48h	0.64
AFL 48h	0.68
H+AFL 48h	0.61
H+AFL 48h	0.63
H+AFL 48h	0.6
H+AFL 48h	0.67
H+AFL 48h	0.65
H+AFL 48h	0.68
BVZ 48h	0.67
BVZ 48h	0.59
BVZ 48h	0.65
BVZ 48h	0.7
BVZ 48h	0.75
H+BVZ 48h	0.57
H+BVZ 48h	0.63
H+BVZ 48h	0.68
H+BVZ 48h	0.7
Ct 72h	0.65
Ct 72h	0.76
Ct 72h	0.84
Ct 72h	0.83
Ct 72h	1.07

Ct 72h	1.02
Ct 72h	1.08
Ct 72h	0.99
Ct 72h	1.11
H 72h	0.6
H 72h	0.58
H 72h	0.69
H 72h	0.73
H 72h	0.92
H 72h	0.92
H 72h	0.86
H 72h	0.9
H 72h	0.98
AFL 72h	1
AFL 72h	1.04
AFL 72h	1.12
AFL 72h	1.14
H+AFL 72h	0.84
H+AFL 72h	0.85
H+AFL 72h	0.81
H+AFL 72h	0.77
H+AFL 72h	0.87
BVZ 72h	0.68
BVZ 72h	0.71
BVZ 72h	0.74
BVZ 72h	0.74
BVZ 72h	0.76
H+BVZ 72h	0.63
H+BVZ 72h	0.67
H+BVZ 72h	0.69

*Ct=control; AFL=aflibercept; H=hypoxia;
H+AFL=hypoxia+aflibercept;
BVZ=bevacizumab;
H+BVZ=hypoxia+bevacizumab
